# Pentamethylquercetin (PMQ) reduces thrombus formation by inhibiting platelet function

**DOI:** 10.1038/srep11142

**Published:** 2015-06-10

**Authors:** Ming-Lu Liang, Xing-Wen Da, Ao-Di He, Guang-Qiang Yao, Wen Xie, Gang Liu, Ji-Zhou Xiang, Zhang-Yin Ming

**Affiliations:** 1 Department of Pharmacology, School of Basic Medicine, Tongji Medical College of Huazhong University of Science and Technology, 13 Hangkong Road, Wuhan 430030, China; 2 The Key Laboratory for Drug Target Research and Pharmacodynamic Evaluation of Hubei Province, Wuhan 430030, China; 3 The Institute of Brain Research, Huazhong University of Science and Technology, 13 Hangkong Road, Wuhan 430030, China

## Abstract

Flavonoids exert both anti-oxidant and anti-platelet activities *in vitro* and *in vivo*. Pentamethylquercetin (PMQ), a polymethoxylated flavone derivative, has been screened for anti-carcinogenic and cardioprotective effects. However, it is unclear whether PMQ has anti-thrombotic effects. In the present study, PMQ (20 mg/kg) significantly inhibited thrombus formation in the collagen- epinephrine- induced acute pulmonary thrombosis mouse model and the ferric chloride-induced carotid injury model. To explore the mechanism, we evaluated the effects of PMQ on platelet function. We found that PMQ inhibited platelet aggregation and granule secretion induced by low dose agonists, including ADP, collagen, thrombin and U46619. Biochemical analysis revealed that PMQ inhibited collagen-, thrombin- and U46619-induced activation of Syk, PLCγ2, Akt, GSK3β and Erk1/2. Therefore, we provide the first report to show that PMQ possesses anti-thrombotic activity *in vivo* and inhibited platelet function *in vitro*, suggesting that PMQ may represent a potential therapeutic candidate for the prevention or treatment of thrombotic disorders.

Thrombotic diseases, such as acute myocardial infarction, acute pulmonary embolism and ischemic stroke, have become the main cause of death in the modern world. It is well established that platelets play a central role in thrombotic diseases^1^. Platelets rapidly adhere to the subendothelial matrix as a result of endothelial cell injury and aggregate with each other to form hemostatic thrombi that prevent blood loss and maintain vascular integrity. This function must be tightly regulated because dysregulated thrombus formation causes blockage of blood vessels, which leads to ischemia^2^.

Flavonoids are polyphenolic compounds naturally present in plants. They were discovered in the 1930s and initially considered to be vitamins because of their effects on capillary permeability. However, epidemiological studies in the 1990s showed that a greater intake of flavonoids was associated with a reduced incidence of cancer and cardiovascular disease^3^. Two mechanisms have been proposed to explain this protective effect, namely the inhibition of low density lipoprotein (LDL) oxidation and the inhibition of platelet aggregation^4^. Quercetin, a flavonoid compound, is widely distributed in plants and present in several fruits and vegetables^3^. Solid evidence showed that, *in vitro*, quercetin exerts anti-oxidant effects, a protective effect on nitric oxide and endothelial function under conditions of oxidative stress and the inhibition of platelet aggregation and LDL oxidation^3^. Rechner *et al.* demonstrated that dietary polyphenolic compounds inhibits platelet aggregation^5^, and Hubbard *et al.* reported that following ingestion of quercetin, collagen-induced platelet aggregation was inhibited^6^. Furthermore, previous studies have shown that the ingestion of quercetin-rich foods and beverages reduced the platelet aggregation caused by different agonists^7^,^8^,^9^.

Although quercetin exhibits a wide range of useful pharmacological properties, there are still many problems with the application of quercetin, such as, low oral bioavailability, a short elimination half-life and low titer. These shortcomings restrict its further development and utilization^10^. During metabolic transformation, the structural modification of quercetin had a profound effect on bioactivity^11^. Specifically, the full methylation of dietary flavonoids results not only in a dramatic increase in their hepatic metabolic stability but also in great improvement of their intestinal absorption, both of which should greatly increase their oral bioavailability^12^,^13^. Bernice *et al.* found that the metabolites of methylated quercetin have a greater ability to penetrate cells than those of quercetin^10^,^14^. According to previous studies, the bioavailability and metabolic stability of a methylated form of quercetin, 3,3',4',5,7-pentamethylquercetin (PMQ) (Fig. 1) were more desirable than those of quercetin^15^.

PMQ is a typical member of the polymethoxylated flavones family^16^. Substantial evidence has emerged demonstrating the health benefits of PMQ, including anti-carcinogenic and cardioprotective properties^15^. However, no studies have assessed its anti-thrombotic and anti-platelet effects.

Therefore, the present study aimed to investigate the effects of PMQ on thrombosis and platelet function. Moreover, we reveal the potential effects of PMQ on the collagen-, thrombin- and U46619-stimulated platelet signaling pathways.

## Results

### Effect of PMQ on collagen-epinephrine-induced acute pulmonary thrombosis in mice

To investigate the effect of PMQ on thrombosis *in vivo*, the collagen-epinephrine-induced acute pulmonary thrombosis mouse model, in which platelet activation is induced by tail vein infusion of collagen and epinephrine, was set up as previously described^17^. As shown in Fig. 2A, intravenous injection of a mixture of collagen and epinephrine resulted in a mortality rate of 100% in the model mice. Treatment with 50 mg/kg aspirin had an anti-thrombotic effect, and 90% of the challenged mice survived. PMQ was also effective in preventing thromboembolic death. However, the survival rates of the animals did not differ significantly when the dose of PMQ was increased from 10 mg/kg to 20 mg/kg. Histological examination of lung tissue from model and vehicle animals revealed platelet thrombi throughout the pulmonary vasculature (Fig. 2B). In contrast, the lungs of mice injected with either 10 mg/kg PMQ, 20 mg/kg PMQ, or 50 mg/kg aspirin were largely devoid of platelet thrombi.

### Effect of PMQ on ferric chloride-induced arterial thrombosis *in vivo*

To further investigate the effects of PMQ on platelet-mediated thrombosis *in vivo*, blood flow through the carotid artery in C57BL/6 mice was measured following ferric chloride injury. According to the above results, we tested vehicle, 10 mg/kg PMQ, 20 mg/kg PMQ and 50 mg/kg aspirin. In model mice and vehicle-treated mice, nearly complete vessel occlusion occurred within the first 12 min following ferric chloride-induced carotid artery damage (Fig. 3A,B). As expected, aspirin 50 mg/kg (positive control) maintained blood flow at levels almost the same as pre-injury levels; similar blood flow levels were observed with 20 mg/kg PMQ (Fig. 3D,E). When we calculated carotid blood flow after ferric chloride injury, we found that 10 mg/kg PMQ, 20 mg/kg PMQ, and 50 mg/kg aspirin significantly improved arterial blood flow during the 12 min following injury compared with the vehicle group (Fig. 3F). These results demonstrate that PMQ prevents thrombosis *in vivo*.

### PMQ inhibits human platelet aggregation induced by various agonists

As platelets have a central role in thrombosis, we investigated the consequences of PMQ treatment on platelet function and performed *in vitro* aggregation studies. PMQ exhibited a dose-dependent inhibitory effect on platelet aggregation stimulated by ADP (2 μM) and collagen (0.5 μg/ml) in human platelet -rich plasma (PRP) (Fig. 4A). Moreover, in washed human platelets, PMQ also exhibited a dose-dependent inhibitory effect on platelet aggregation stimulated by collagen (0.5 μg/ml), thrombin (0.04 U/ml) and U46619 (0.3 μM) (Fig. 4B). Pretreatment of platelets with 40 μM PMQ almost completely abolished collagen- and thrombin-induced platelet aggregation (Fig. 4B).

### PMQ inhibits collagen- and thrombin-induced platelet granule secretion

Platelets contain dense and alpha granules that release their contents in response to stimulation with agonists to amplify platelet activation, which facilitates thrombosis both *in vitro* and *in vivo*. To investigate whether PMQ also has an inhibitory effect on granule secretion, washed human platelets were preincubated with various concentrations of PMQ for 5 min at 37 °C and stimulated with collagen and thrombin. We found that dense granules secretion induced by collagen and thrombin was significantly inhibited by PMQ (Fig. 5A). P-selectin expression was measured to analyze alpha granules secretion by flow cytometry (Fig. 5B). Consistent with the dense granules secretion results, we found that P-selectin expression induced by collagen and thrombin was also significantly reduced by PMQ. These data demonstrate that pretreatment of platelets with PMQ significantly inhibited platelet granule secretion.

### PMQ inhibits collagen-, thrombin- and U46619-stimulated platelet aggregation signaling pathways

To investigate the signaling events by which PMQ inhibited collagen, we analyzed the phosphorylation of several molecules downstream of the GPVI/α2β1 signaling pathway, including Syk, PLCγ2 and MAPKs, and found that collagen-stimulated Syk and PLCγ2 phosphorylation was significantly inhibited by treatment with 40 μM PMQ. We also investigated the effect of PMQ on the MAPKs signaling pathways downstream of collagen/GPVI signaling. Collagen stimulation led to the activation of all three MAPKs (p38, Erk1/2 and JNK1/2) in platelets, but only Erk1/2 phosphorylation was inhibited by PMQ (Fig. 6A). We analyzed thrombin- and U46619-induced platelet activation, mediated by the G_q_ signaling pathway, and found that the inhibition effect of Erk1/2 phosphorylation was similar to that seen with collagen (Fig. 6B,C). PMQ also inhibited the thrombin- and U46619-induced Syk, PLCγ2 expression.

PI3K plays an important role in platelet activation. Therefore, to further reveal the underlying molecular mechanisms of the inhibitory effects of PMQ on platelet activation, we conducted biochemical analysis with platelet lysates. Pretreatment of platelets with PMQ prevented Akt, an effector molecule downstream of PI3K signaling, from being phosphorylated in platelets challenged with collagen (Fig. 6). Similarly, collagen-induced phosphorylations of GSK3β, a substrate of Akt, was attenuated by PMQ. Akt and GSK3β phosphorylation was also decreased in platelets activated by thrombin and U46619. These findings suggest that PMQ functions as a negative regulator of PI3K/Akt signaling to inhibit agonist-induced platelet activation by acting on multiple signaling pathways to suppress platelet function.

## Discussion

In the present study, we observed that PMQ exerts anti-thrombotic activity *in vivo*. PMQ increased the survival rate in mice after collagen-epinephrine-induced acute pulmonary thrombosis and reduced platelet thrombi. In addition, PMQ increased carotid blood flow after ferric chloride-induced injury and extended the occlusion time. These results indicate that PMQ has an anti-thrombotic effect *in vivo*.

Because of the central role of platelets in arterial thrombosis, anti-platelet therapy is beneficial in thrombosis-related diseases. Therefore, we investigated the effects of PMQ on platelets and found that PMQ inhibited ADP-, collagen-, thrombin-, and U46619-induced platelet aggregation. PMQ also inhibited both the release of dense granules and the secretion of alpha granules. Granule secretion is critical to the amplification of platelet activation, the recruitment of circulating platelets into aggregates, and thrombus stabilization.

Biochemical studies revealed that PMQ treatment inhibited collagen-induced phosphorylation of molecules involved in the GPVI-Syk-PLCγ2-MAPKs and PI3K-Akt-GSK3β signaling pathways. Collagen/GPVI initiates Src family kinase (SFK)-based signaling that leads to the activation of Syk/PLCγ2^18^,^19^,^20^,^21^. Our findings that PMQ inhibited collagen-induced platelet aggregation and secretion indicate that PMQ may inhibit collagen/GPVI signaling. However, PI3K signaling also contributes to collagen/GPVI-mediated activation of PLCγ2 by providing PIP3, to which PLCγ2 binds and translocates to the membrane^22^. The translocation of PLCγ2 to the membrane from the cytosol is essential for its full activation^23^. Akt is a downstream effector of PI3K, and a well-accepted marker of the activation of the PI3K/Akt signaling pathway^24^. The role of Akt and its substrate GSK3β in platelet functioning have been addressed in inhibitor and genetic studies^25^,^26^,^27^. Thrombin and U46619 stimulate platelet activation mainly through the Gq pathway. Gq proteins activate PLCβ leading to calcium mobilization (via inositol-1,4,5-trisphosphate) and activation of protein kinase C (PKC) (via DAG). Gq proteins also activate the PI3K/Akt pathway^28^. Our data, which showed that PMQ suppressed collagen-, thrombin- and U46619-induced phosphorylation of Akt and GSK3β, support the notion that PMQ also functions, at least in part, as an inhibitor of the PI3K/Akt pathway to exert its inhibitory effects on platelet aggregation. Our study also found that PMQ inhibited collagen-, thrombin- and U46619-induced phosphorylation of Erk1/2; therefore, in addition to functioning as an inhibitor of GPVI-Syk-PLCγ2-Erk1/2 signaling, PMQ also acts as a repressor of PI3K-Akt-GSK3β signaling to attenuate platelet activation. Moreover, PMQ also inhibited collagen-, thrombin- and U46619-induced phosphorylation of Syk and PLCγ2. Taken together, these data indicated that PMQ remarkably impaired integrin αIIbβ3 “outside-in” signaling-related platelet function.

Wright, B proved that a group of anti-oxidants in the flavonoid family inhibits platelet aggregation *in vitro*^29^ and we got the same result. Hubbard, G. P. *et al* also suggest that a diet high in flavonoids may inhibit platelet aggregation *in vivo*^6^, the antithrombotic effect of PMQ approve this notion. Our western blot results that PMQ strongly inhibit Syk (Y525/526) and PLCγ2(Y1217) is consistent with Wright, B^14^. We extended the study *in vivo* and found that PMQ inhibits thrombus formation in acute animal model. Our study showed for the first time that PMQ participates in a number of intracellular events associated with platelet activation and thrombosis. Our data provide an insight for the chemical modification of flavonoids for use as anti-platelet agents. Nevertheless, our experiment was associated with limitations. For example, we did not include other flavonoid compounds as comparators.

In summary, this study is the first to report that PMQ protected mice from death in the acute lung thromboembolism model and from carotid artery injury induced by ferric chloride and that PMQ inhibited platelet aggregation induced by several agonists. In addition, we show that PMQ regulated functional responses in platelets, including the release of ATP and P-selectin. Finally, we show that the underlying molecular mechanisms of the inhibitory effects of PMQ on platelet function appear to be the suppression of the PI3K/Akt-GSK3β and Syk-PLCγ2-Erk signaling cascades. Besides, the effect of PMQ on endothelial cells and white blood cells may contribute to its anti-thrombosis. Therefore, our results suggest that PMQ has a potent inhibitory effect on platelet function. However, such high concentrations would unlikely be reached in circulation from intake of food and even from diet supplements. So PMQ may be considered as a potential therapeutic agent for abnormal platelet activation-related diseases, such as thrombosis and arteriosclerosis.

## Methods

### Study subjects

Healthy volunteers without a history of hematological diseases (such as platelet and coagulation disorder) who did not take any drugs (including cigarettes) that may affect platelet function in the preceding 2 weeks were recruited for this study. Male and female volunteers (aged 20–35 years) were recruited from the staff and students at the Tongji Medical College, Huazhong University of Science and Technology. Each volunteer provided written informed consent prior to the collection of 30 ml of blood. All of the experimental procedures were approved by the Ethics Committee of Huazhong University of Science and Technology strictly in accordance with Helsinki declaration for the Use of Human Subjects.

Male C57BL/6 mice (18–22 g) were provided by the Experimental Animal Center of Tongji Medical College, HUST. All of the animals were maintained in a standard laboratory animal facility with free access to feed and water and acclimated for at least 2 weeks before use. The study protocol was reviewed and approved by the Ethics Committee for experimental animals, Tongji Medical college (no.13, Hangkong Road, Wuhan, P.R. China, IACUC no. 302) and in accordance with the ARRIVE guidelines^30^.

### Reagents

**Figure 1:**
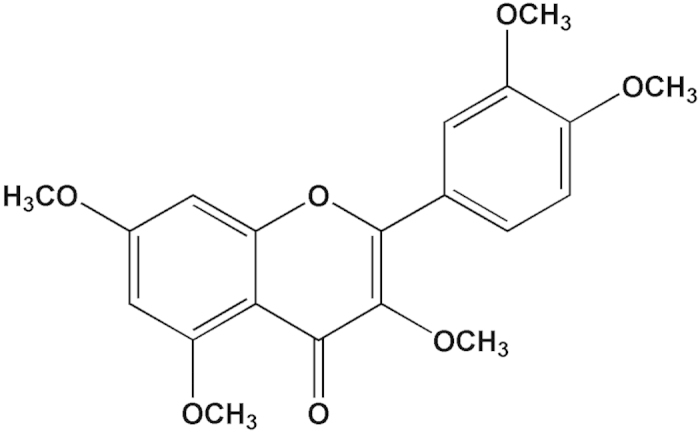
Chemical structure of 3,3',4',5,7-pentamethylquercetin (PMQ).

**Figure 2:**
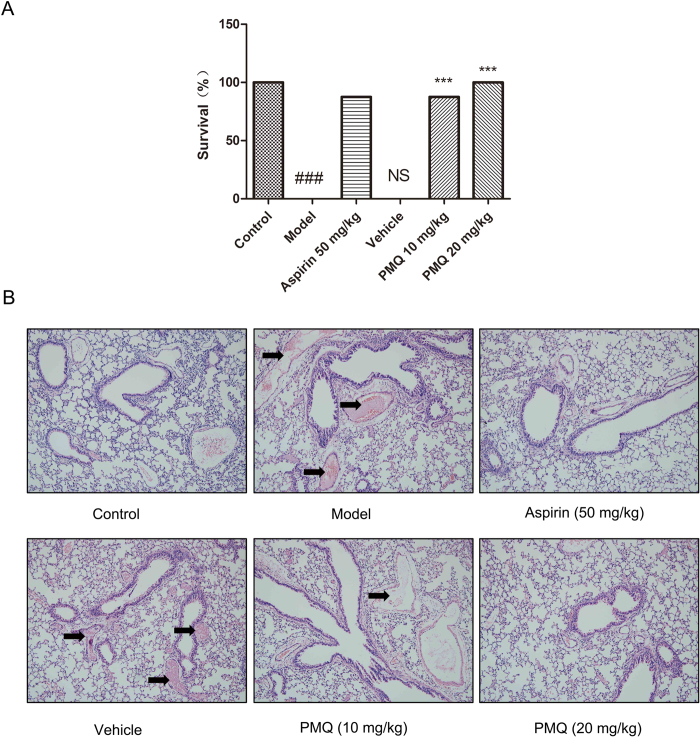
PMQ- or aspirin-treated mice were protected from microvascular thrombosis. Mice were pre-treated with vehicle, 10 mg/kg PMQ, 20 mg/kg PMQ, or 50 mg/kg aspirin for 30 min prior to injury with intravenous injection of collagen (3.57 mg/kg) and epinephrine (0.143 mg/kg). (**A**) PMQ was effective in preventing collagen-epinephrine-induced thromboembolic death (n = 10). *** p < 0.001 vs. vehicle, ### p < 0.001 vs. control, NS not significant vs. model. (**B**) Representative sections of H&E-stained lung sections from mice treated as labeled. Magnification: ×200.

**Figure 3:**
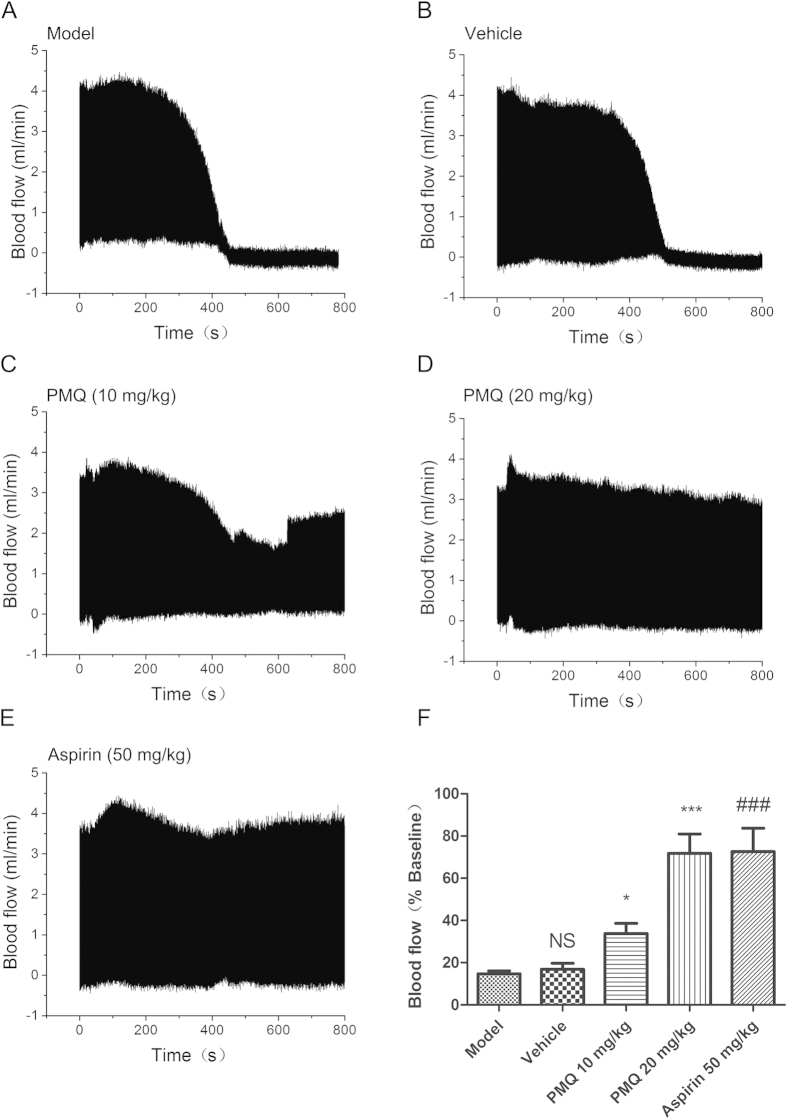
PMQ- or aspirin-treated mice were protected from ferric chloride-induced carotid arterial injury. (**A**–**E**) Blood flow was measured at 12 min after arterial injury. Mice were treated with vehicle, 10 mg/kg PMQ, 20 mg/kg PMQ, or 50 mg/kg aspirin (positive control) by tail vein injection 30 min prior to arterial injury. (F) Quantification of blood flow. Data are expressed as the mean ± SEM of 10 experiments. * p < 0.05, *** p < 0.001 vs. vehicle, ### p < 0.001 vs. model, NS not significant vs. model.

**Figure 4:**
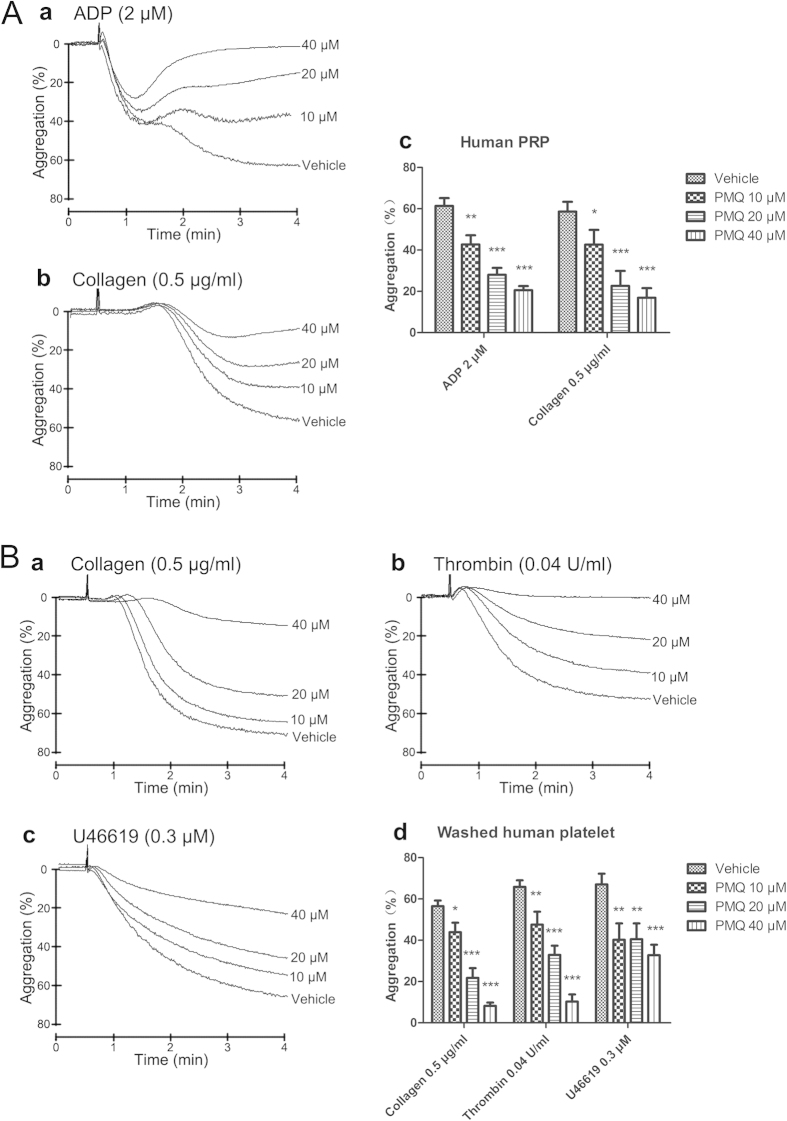
Inhibitory effect of PMQ on agonist-induced platelet aggregation in human PRP and human washed platelets. (**A**) PRP (3.0 × 10^8^/ml) were pre-incubated with PMQ (10 μM, 20 μM, and 40 μM) or a solvent control before the addition of ADP (2 μM) or collagen (0.5 μg/ml) to trigger platelet aggregation. Quantification of aggregation (%) was also shown in the right panel. (**B**) Washed platelets (3.0 × 10^8^/ml) were pre-incubated with PMQ (10 μM, 20 μM, and 40 μM) or a solvent control prior to the addition of collagen (0.5 μg/ml), thrombin (0.04 U/ml), or U46619 (0.3 μM) to trigger platelet aggregation. Quantification of aggregation (%) was also shown in the lower panel. Data are expressed as the mean ± SEM of four experiments. * p < 0.05, ** p < 0.01, *** p < 0.001 vs. vehicle.

**Figure 5:**
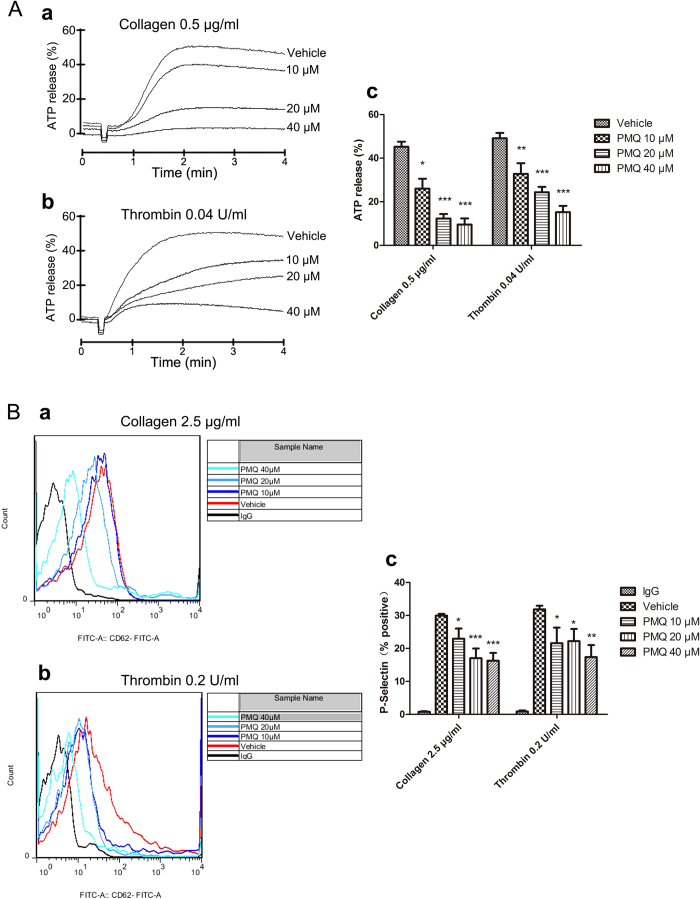
Inhibitory effect of PMQ on agonist-induced ATP-release and surface P-selectin expression in washed human platelets. (**A**) Washed platelets (3.0 × 10^8^/ml) were pre-incubated with PMQ (10 μM, 20 μM, and 40 μM) or a solvent control followed by the addition of collagen (0.5 μg/ml) or thrombin (0.04 U/ml) in the ATP-release reaction experiment. Quantification of ATP release (%) was also shown in the right panel. (**B**) Washed human platelets were pre-incubated with PMQ (10 μM, 20 μM, and 40 μM) at room temperature for 5 min; FITC-conjugated P-selectin was added 5 min after collagen (2.5 μg/ml) or thrombin (0.2 U/ml) stimulation. P-selectin expression was determined by flow cytometry and presented as representative overlay histograms. Quantification of P-selectin expression (%) was also shown in the right panel. Representative overlay histograms were composed with FlowJo version 7.6 (Tree Star Inc., Ashland, OR, USA). The results are expressed as the mean ± SEM of four experiments. * p < 0.05, ** p < 0.01, *** p < 0.001 vs. vehicle.

**Figure 6:**
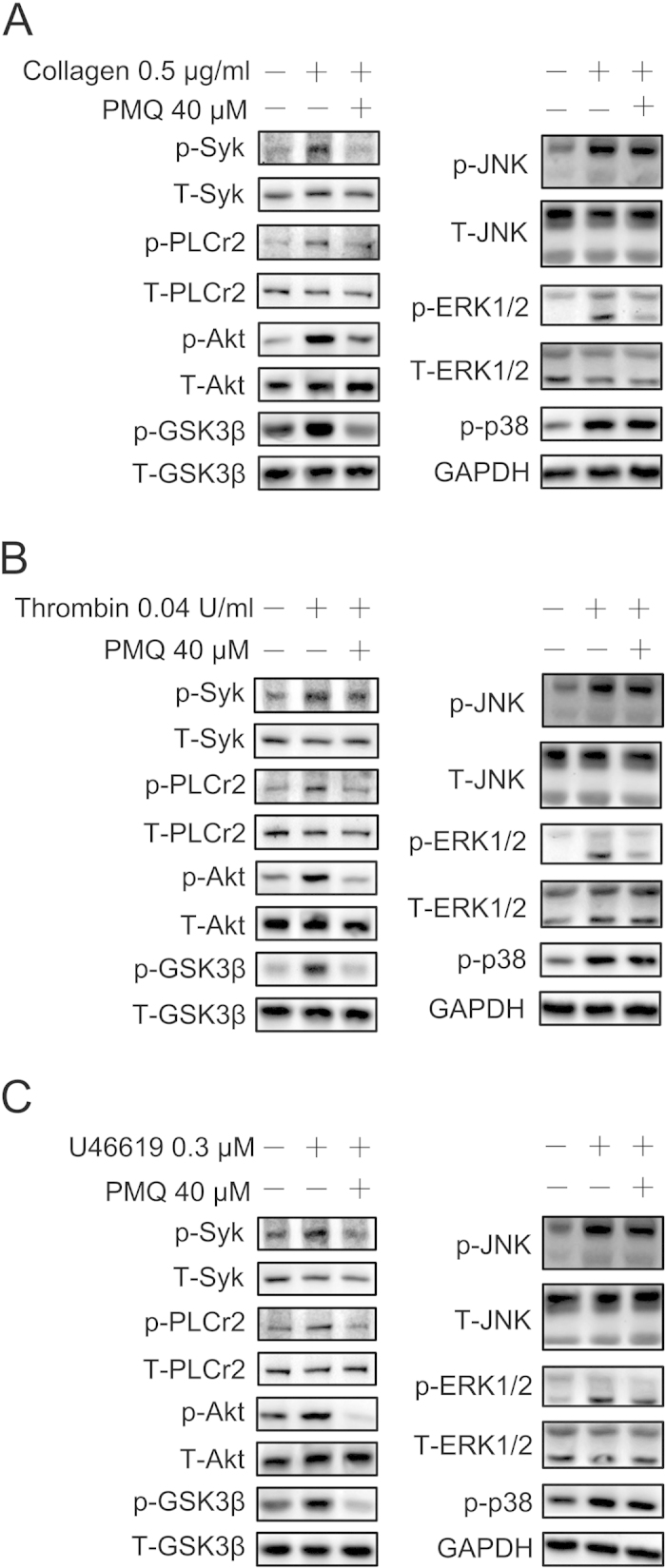
Effect of PMQ on agonist-induced signal transduction in washed human platelets. Washed platelets (3 × 10^8^/ml) were stimulated with (**A**) 0.5 μg/ml collagen, (**B**) 0.04 U/ml thrombin, and (**C**) 0.3 μM U46619 for 180 s and subsequently lysed with cell lysis buffer. Proteins were separated by SDS-PAGE and transferred to a PVDF membrane. The membrane was probed with the relevant antibodies. Staining of the respective total proteins or GAPDH served as loading controls, all the gels have been run under the same experimental conditions.

PMQ (3,3′,4′,5,7-pentamethylquercetin, HPLC grade with ≥98% purity; Fig. 1) was synthesized at the laboratory of Prof. Jin in our department^31^. Thrombin and U46619 were obtained from Sigma (St. Louis, MO, USA). Collagen and luciferin-luciferase was purchased from Chrono-Log Corp (Havertown, PA, USA). Antibodies against total JNK, phospho-JNK (Thr183/185), total-AKT, total-Syk, total-PLCγ2, total-Erk, phospho-Erk (Tyr 204), total-GSK3β were obtained from Santa Cruz Biotechnology (Santa Cruz, CA, USA). Antibodies against phospho-p38 (Thr180/Tyr182), phoshpo-PLCγ2 (Tyr1217), phospho-Syk (Tyr525/526), phospho-AKT (Ser473), and phospho-GSK3β (Ser9) were obtained from Cell Signaling (Beverly, MA, USA). Anti-CD62P (P-selectin) antibodies were obtained from BD Biosciences (San Jose, CA, USA). The ECL western blotting detection reagent was obtained from Pierce Chemical Co (Rockford, Illinois, USA). 2.5 mM, 5 mM, 10 mM PMQ stock solution was dissolved in 80% dimethylsulfoxide (DMSO) and stored at room temperature until use, 1 μl stock solution was further diluted in 250 μl platelet suspension *in vitro* experiment. And PMQ was dissolved in dilution buffer ( polyethyleneglycol: Glycerin: physiological saline = 4 : 2 : 9) *in vivo* experiment.

### Platelet preparation

Human platelets were prepared as previously described^17^,^32^. Human blood was drawn from the cubital vein without stasis into siliconized vacuum containers containing 1:9 (v/v) 3.8% sodium citrate. PRP was obtained by centrifuging whole anti-coagulant blood for 10 min at 150 × g. Platelets were pelleted by centrifuging the PRP at 800 × g for 10 min. The supernatant was allocated to a platelet-poor plasma (PPP) fraction, which was used as a reference solution in the aggregation assays. Platelets were washed with Tyrode’s buffer (137 mM NaCl, 13.8 mM NaHCO_3_, 5.5 mM glucose, 2.5 mM KCl, 20 mM HEPES, 0.36 mM NaH_2_PO_4_, pH 7.4) containing 1 μM PGE1 and 2.5 mM EDTA and finally resuspended in Tyrode’s buffer not containing PGE1 or EDTA to a final concentration of 3.0 × 10^8^ platelets/ml. All of the platelet preparations were conducted at room temperature. The resuspended platelet standed at least 30 minutes before experiment.

### Platelet aggregation and ATP release assay

Platelet aggregation and ATP release assays were performed as described previously^17^ with an aggregometer (Chrono-Log, Havertown, PA, USA). The platelet pellet was resuspended in Tyrode-HEPES buffer and the concentration was adjusted to 3 × 10^8^/ml for use in the aggregation studies. CaCl_2_ (1 mM) was added prior to agonist stimulation. The PRP and washed platelets were pre-incubated at 37 °C for 5 min with either PMQ alone or vehicle, then stimulated with different agonists, while stirring, for 3 min at 37 °C, and aggregation trace was recorded^33^. Platelet secretion was determined by measuring the release of ATP using the luciferin-luciferase reagent (Chrono-lume, Chrono-Log, USA). And the quantification was got from the actual aggregation and release tracings.

### Flow cytometric analysis

Flow cytometric analysis was performed as described previously^34^. Platelets were pre-incubated with PMQ (10, 20 and 40 μM) or vehicle for 5 min prior to incubation with collagen (2.5 μg/ml) and thrombin (0.2 U/ml) for 5 min at 37 °C. The binding of FITC-conjugated anti-CD62P antibodies to human platelets (5 × 10^7^/ml) was conducted by incubation in the dark at room temperature for 15 min and analyzed with a BD Biosciences flow cytometer (San Jose, CA, USA).

### Collagen-epinephrine-induced acute pulmonary thrombosis in mice

To investigate the effect of PMQ on thrombosis *in vivo*, the collagen-epinephrine-induced acute pulmonary thrombosis mouse model was set up as previously described^35^. Mice were grouped as control, vehicle, 50 mg/kg aspirin, 10 mg/kg PMQ and 20 mg/kg PMQ, and these treatments were administered via the enterocoelia vein 30 minutes prior to injury. All of the groups except for control were injected with a mixture of collagen (3.57 mg/kg) and epinephrine (0.143 mg/kg) in the tail vein to induce thrombus formation *in vivo*. The survival rate in each group was determined within 5 min and lung sections were visualized using H&E staining.

### Ferric chloride-induced carotid injury model

Ferric chloride-induced arterial injury was performed as previously described^36^,^37^. Briefly, mice were anesthetized with urethane by intraperitoneal injection. A midline cervical incision was made and the carotid artery exposed. A Doppler flow probe (Transonic, TS420, UK) was placed proximal to the carotid artery to measure baseline blood flow. A 2-mm strip of filter paper saturated with 10% ferric chloride was applied to the carotid artery on the adventitial surface of the vessel for 3 min. Following the removal of the filter paper, blood flow through the carotid artery was monitored for 12 min, or until vessel occlusion reached 95%. Area under the curve (AUC) and carotid artery blood flow at 12 min were recorded. At the end of each experiment and whilst under deep anesthesia, the mice were euthanized by cervical dislocation.

### Immunoblotting

For aggregation, after incubation with vehicle or 40 μM PMQ for 5 min, washed human platelets were stimulated with or without agonists for 3 min. Aggregated platelets were lysed by adding the same volume of 2 × lysis buffer (30 mM HEPES, 300 mM NaCl, 20 mM EGTA, 0.2 mM MgCl_2_, 2% Triton X-100, 2 × protease and 2 × phosphatase inhibitor cocktails) into the reactions. The resulting protein concentrations were quantified using the Enhanced BCA protein assay kit (Beyotime Inc., China). Western blot analysis was performed as described previously^38^. Equal amounts of protein were fractionated via SDS-PAGE and transferred to a PVDF membrane, blocked with 5% BSA in TBST (50 mM Tris, pH 7.5, 250 mM NaCl, 0.2% Tween 20) and probed with antibodies overnight at 4 °C. The membranes were washed in TBST three times and exposed to the appropriate secondary antibodies for 1 h at room temperature. Immunoreactive bands were visualized using DNR bio-imaging systems according to the manufacturer’s instructions and quantified by Image J.

### Statistical analysis

Data were analyzed using SPSS Statistics version 19 for Windows (IBM, New York, USA) and presented as the mean ± standard error. Data were assessed by analysis of variance. A two-tailed unpaired Student’s t test was used to determine whether the differences between groups were significant. The mice survival rate was evaluated by using a chi-square test. *p* values less than 0.05 were considered to be statistically significant.

## Additional Information

**How to cite this article**: Liang, M.-L. *et al.* Pentamethylquercetin (PMQ) reduces thrombus formation by inhibiting platelet function. *Sci. Rep.* **5**, 11142; doi: 10.1038/srep11142 (2015).
